# BRCA1 overexpression attenuates breast cancer cell growth and migration by regulating the pyruvate kinase M2-mediated Warburg effect *via* the PI3K/AKT signaling pathway

**DOI:** 10.7717/peerj.14052

**Published:** 2022-09-28

**Authors:** Xiuli Liu, Hanxu Liu, Lei Zeng, Yuetao Lv

**Affiliations:** Department of Thyroid and Breast Surgery, Jining No.1 People’s Hospital, Jining, Shandong, China

**Keywords:** *BRCA1*, PKM2, Breast cancer cell, Glycolysis, PI3K/Akt

## Abstract

This work explored the mechanism of the effect of breast-cancer susceptibility gene 1 (*BRCA1*) on the metabolic characteristics of breast cancer cells, including the Warburg effect and its specific signaling. We transfected MCF-7 cells with a *BRCA1*-encoding LXSN plasmid or PKM2 siRNA and examined cancer cell metabolism using annexin V staining, inhibitory concentration determination, Western blotting, glucose uptake and lactic acid content measurements, and Transwell assays to assess glycolytic activity, cell apoptosis, and migration, and sensitivity to anti-cancer treatment. The *BRCA1*-expressing MCF-7 cells demonstrated low PKM2 expression and decreased glycolytic activity (downregulated hexokinase 2 (HK2) expression, upregulated isocitrate dehydrogenase 1 (IDH1) expression, and reduced O_2_ and glucose consumption and lactate production) *via* regulation of PI3K/AKT pathway compared with the empty LXSN group. *BRCA1* transfection slightly increased apoptotic activity, decreased cell migration, and increased the IC_50_ index for doxorubicin, paclitaxel, and cisplatin. Inhibiting PKM2 using siRNA attenuated the IC_50_ index for doxorubicin, paclitaxel, and cisplatin compared with the control. Inhibiting PKM2 activated PI3K/AKT signaling, increased apoptosis, and decreased MCF-7 cell migration. Our data suggest that *BRCA1* overexpression reverses the Warburg effect, inhibits cancer cell growth and migration, and enhances the sensitivity to anti-cancer treatment by decreasing PKM2 expression regulated by PI3K/AKT signaling. These novel metabolic findings represent a potential mechanism by which *BRCA1* exerts its inhibitory effect on breast cancer.

## Introduction

Cancer cells are hypermetabolic and rely heavily on “aerobic glycolysis”, which is demonstrated to be the metabolic hallmark of cancer cell (Li et al., 2020a). The conversion of glucose to lactate, which occurs under hypoxic condition in normal cellular environment, is exhibited in cancer cells despite the presence of oxygen, which normally suppresses glycolytic activity. Aerobic glycolysis, known as the Warburg effect, is one of the main metabolic signatures of cancer cells. Recent work have demonstrated that continuous aerobic glycolysis in cancer cell triggers oncogene development or the loss of tumor-suppressor genes. Recent discoveries have identified tumor-suppressor genes related to metabolic pathways, including citrate metabolism, such as *sdha/c* in paragangliomas and *idh1/2* in gliomas (Thompson, 2009). Oncogenes and tumor-suppressor genes regulate cancer metabolism, reflecting the importance of discovering metabolic biomarkers and therapeutic targets (Morvan & Demidem, 2007). Breast-cancer susceptibility gene 1 (*BRCA1*) is a major tumor-suppressor gene associated with several anti-carcinogenic pathways. *BRCA1* is downregulated in many sporadic cases of breast cancer (Miki et al., 1994; Narod & Foulkes, 2004). The molecular profiles of *BRCA1*-mutation breast cancer are featured by the lack of expression of estrogen receptor, progesterone receptor and human epidermal growth factor receptor 2 (Yin et al., 2020). Immunohistochemical profiles of the *BRCA1*-mutation breast cancer are featured by expression of cytokeratin (CK) 5/6 and CK14, contributing to the increasing risk of breast cancer (Rigakos & Razis, 2012). *BRCA1* affects the cellular response to stress and senses DNA damage, as well as participates in DNA repair, cell cycle regulation, transcription, ubiquitination, apoptotic activity, and resistance to anticancer drugs (Yarden & Papa, 2006).

In the Warburg effect, pyruvate kinase M2 (PKM2) regulates metabolic pathways. Increased PKM2 expression in cancer cells can enhance glycolytic activity and provide sufficient energy for the rapid growth of cancer cells. In numerous human cancers, PKM2 expression is higher than that in normal tissues (Liu et al., 2018). Knocking down PKM2 in human hepatocellular carcinoma cells inhibited their function and proliferation and increased apoptosis (Xu et al., 2015).

However, the role and mechanism of *BRCA1* in cancer cell metabolism remain unclear. *BRCA1* has been demonstrated to regulate *de novo* fatty acid synthesis (Moreau et al., 2006), and protect tumor cells against oxidative stress (Bae et al., 2004). *BRCA1* is reported to induce major energetic metabolism reprogramming in breast cancer cells (Privat et al., 2014). To explore this, metabolic and other functional profiles were measured in breast cancer cells expressing *BRCA1*. Furthermore, this work explored the specific signaling pathway of *BRCA1* and whether the glycolytic enzyme PKM2 is involved in the effects of *BRCA1*.

## Materials and Methods

### Biological materials

Michigan Cancer Foundation-7 (MCF-7) human breast cancer cells (No. TCHu 74; cell bank of Typical Culture Preservation Committee of Chinese Academy of Science, Shanghai, China) were incubated in high-glucose Dulbecco Modified Eagle Medium (DMEM, No. 11965092; Gibco, Waltham, MA, USA) supplemented with 10% fetal bovine serum (FBS, No. 16140071; Gibco, Waltham, MA, USA) at 37 °C under 5% CO_2_. MCF-7-*BRCA1* was stably transfected using a *BRCA1*-encoding LXSN plasmid (Shanghai GeneChem Co., Ltd., Shanghai, China) (Privat et al., 2009). While control cells (MCF-7-LXSN cell) were transfected with the LXSN empty vector. The transfection effect was measured for BRCA1 level by Western blotting. All experimental procedures related to the treatment of animals were performed adhere to the Animal Care and Use Committee of Ji Ning First People’s Hospital (JNRM-2022-DW-006).

### Apoptotic activity

The percentage of apoptotic cells was evaluated *via* the Annexin V-PE apoptosis detection kit (BD Pharmingen, Franklin Lakes, NJ, USA). After treatment, the specimens were washed using phosphate buffered saline (PBS), treated with annexin V and propidium iodide for 30 min, and measured using flow cytometry (BD FACS Calibur, San Jose, CA, USA).

### Inhibitory concentration

The MTS assay was used to measure inhibitory concentrations adhere to the instruction of the CellTiter96^®^ Aqueous One Solution Cell Proliferation Assay (Promega, Madison, WI, USA). MCF-7 cells (1 × 10^4^) were planted on 96-well microplates containing DMEM with 1% FBS and LXSN plasmid or siRNA, simultaneously with an anti-cancer agent. The MTS assay was performed after 24 h. The IC_50_ was determined by extrapolating the log concentration *vs* cell viability.

### Western blotting

MCF-7 cells were lysed in rapid immunoprecipitation assay (RIPA) solution with phosphatase and protease inhibitors, and the concentration of proteins was evaluated *via* the bicinchoninic acid (BCA) method. Proteins were separated *via* 8–10% sodium dodecyl sulfate-polyacrylamide gel electrophoresis (SDS-PAGE) gel and then loaded onto the nitrocellulose membrane. The membranes were treated with 5% non-fat milk for 90 min and then treated by the primary antibodies (anti-BRCA-1 (No. ab238983, 1:1,000; Abcam, Cambridge, UK), anti-PKM2 (No. ab85555, 1:1,000; Abcam, Cambridge, UK), anti-hexokinase 2 (HK2) (No. ab209847, 1:1,000; Abcam, Cambridge, UK), anti-isocitrate dehydrogenase 1 (IDH1) (No. ab172964, 1:1,000; Abcam, Cambridge, UK), anti-phospho-AKT(Thr308) (No.#13038, 1:1,000; Cell Signaling Technology, Danvers, MA, USA), anti-AKT (No.#4691, 1:1,000; Cell Signaling Technology, Danvers, MA, USA) and anti-β-actin (No. ab8227, 1:1,000; Abcam, Cambridge, UK)) overnight, then incubated with the secondary antibodies (anti-mouse IgG (No. 7076; 1:1,000; Cell Signaling Technology, Inc., Danvers, MA, USA) or anti-rabbit IgG (No. 7074; 1:1,000; Cell Signaling Technology, Danvers, MA, USA)) for 1 h. Bands were evaluated using an imaging system (Bio-Rad, Hercules, CA, USA).

### Glucose uptake and lactate measurement

Cells were plated in six-well plate (1 × 10^5^/well) at 37 °C under 5% CO_2_ for 1 day. After washing the wells with PBS, the culture medium was replaced with medium without FBS, and then the supernatant was collected. A glucose and lactate kit (Nanjing Jiancheng Bioengineering Institute, Nanjing, China) was applied to measure glucose consumption and lactic acid release.

### Transwell assay

To assess migration, MCF-7 cells were trypsinized and placed in transwell cell culture inserts (Corning Inc., Corning, NY, USA), including non-coated membranes. Then, 1% FBS (600 μL) was added into the lower chamber for 1 day, the upper surface of the membranes was wiped with a cotton tip, and the lower surface were incubated for 30 min using 0.1% crystal violet.

### Small interfering RNA (siRNA)

PKM2-specific siRNA was used to knockdown PKM2. In line with the manufacturer’s instruction, MCF-7 cells were transfected using 100 pmol PKM2 siRNA *via* Lipofectamine 2000 (Life Technologies, Carlsbad, CA, USA). At 6 h after transfection, the medium was changed by medium with 5% FBS for 1 day.

### Statistical analysis

All results (mean ± SD) were analyzed using SPSS 21.0 (SPSS, Chicago, IL, USA). Two groups with normal distributions were compared *via t*-tests. Statistical significance was defined as *P* < 0.05.

## Results

### BRCA1 overexpression decreased PKM2 expression and increased apoptosis in MCF-7 cells

**Figure 1:**
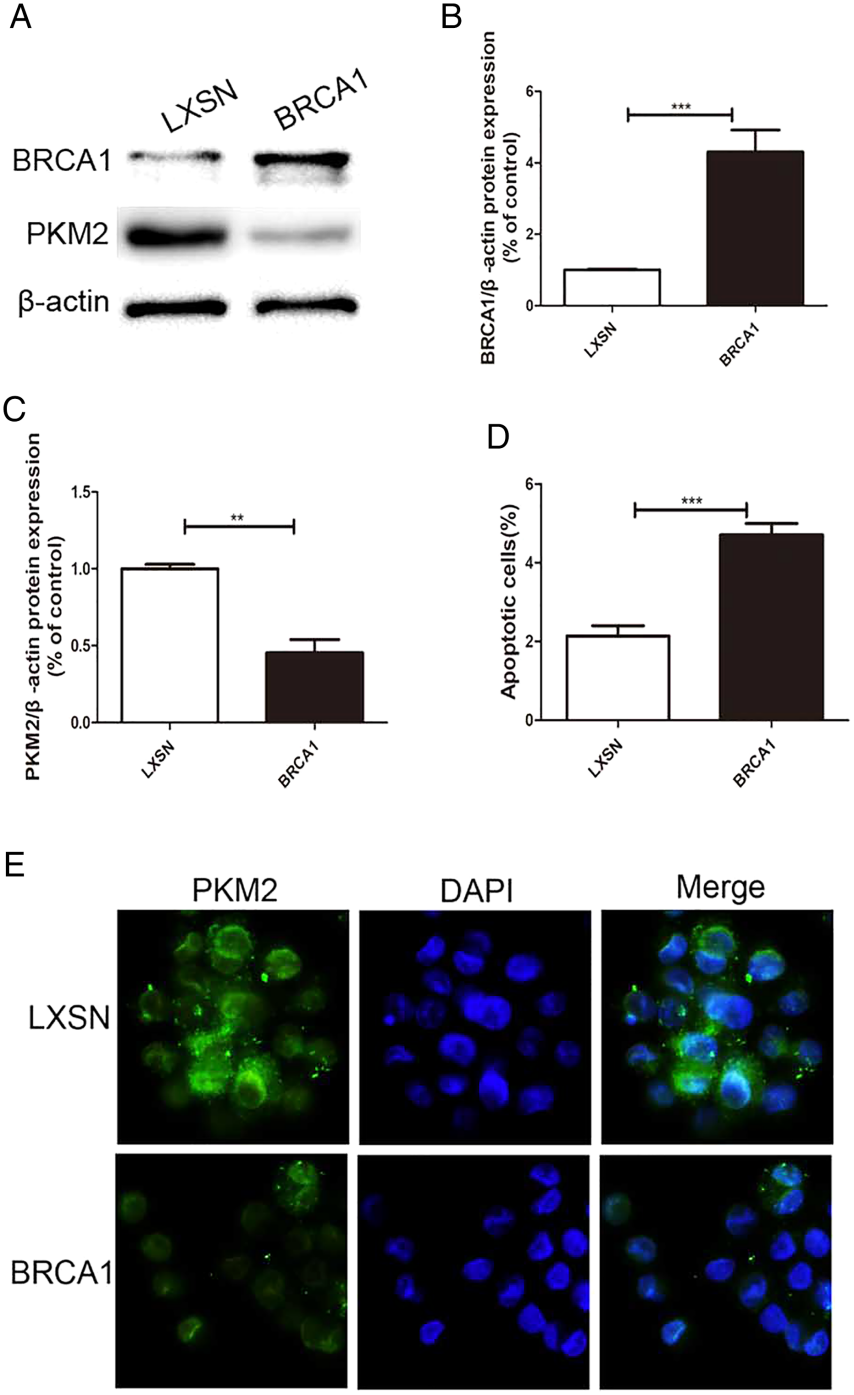
BRCA1 overexpression decreased the expression of PKM2 and increased apoptosis in MCF-7 cell. (A–C) Level of BRCA1 and PKM2 was tested by Western blot and quantification analysis. (D) Number of apoptotic cells was measured at 2 days *via* the annexin V assay. (E) Immunofluorescence imaging of the PKM2 expression and the nucleus (DAPI, blue). Values are represented as mean ± SD, *n* = 3 (**P < 0.01 and ***P < 0.001).

MCF-7 cells were transfected *via* a LXSN plasmid expressing the *BRCA1* protein and showed higher expression of *BRCA1* (Figs. 1A, 1B), while Control cells (MCF-7-LXSN cell) were transfected with the LXSN empty vector. The *BRCA1*-expressing MCF-7 cells exhibited lower PKM2 expression compared with the empty-LXSN group (Figs. 1A–1C), which is consistent with the immunofluorescence results (Fig. 1E). The MCF-7-*BRCA1* group showed slightly enhanced apoptotic activity compared with the MCF-7-LXSN group (Fig. 1D).

### *BRCA1* decreased glycolysis

**Figure 2:**
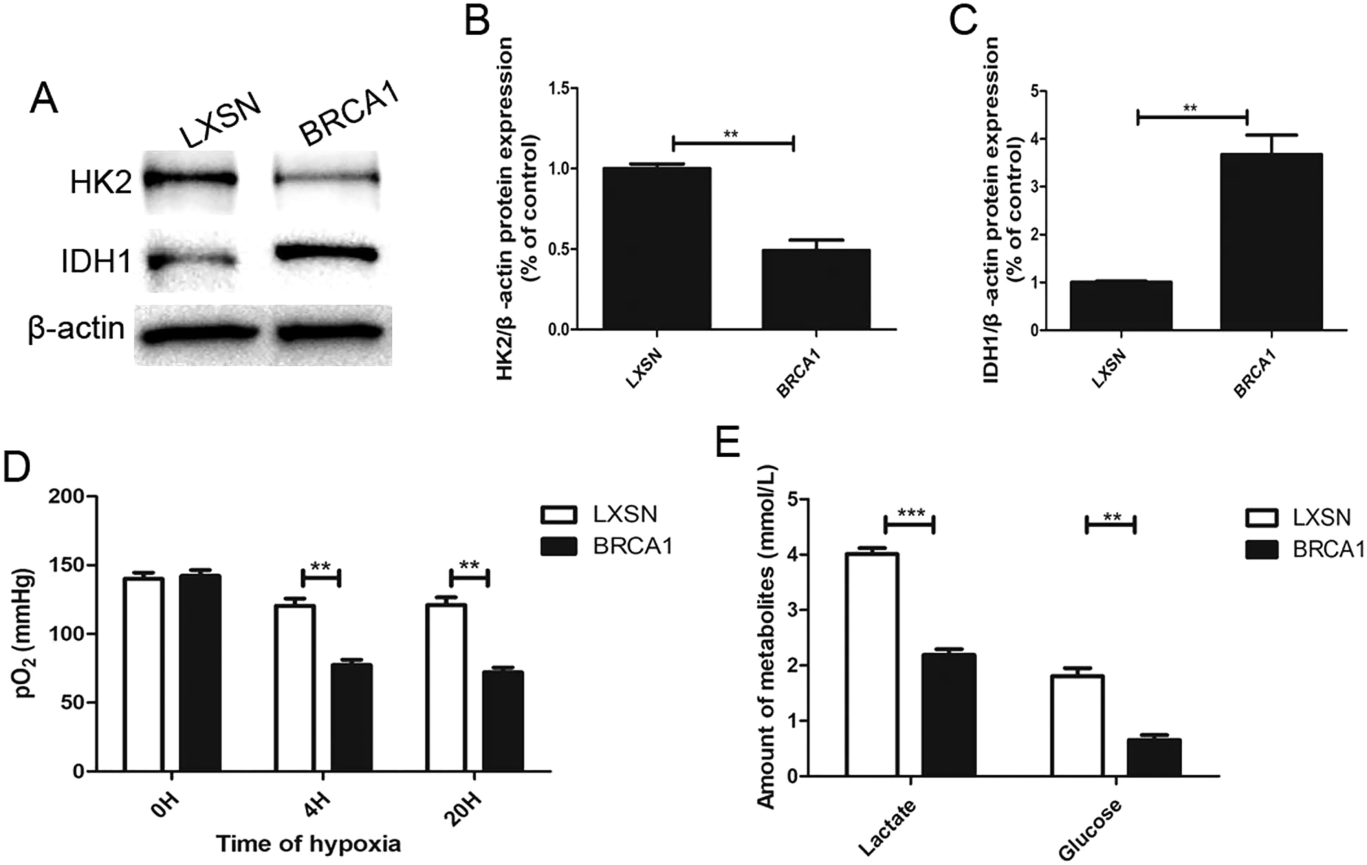
BRCA1 decreased activity of glycolysis. (A–C) Western blotting and quantification analysis of HK2 and IDH1 enzymes. (D) The pO2 was tested in the culture medium under the hypoxic condition for the indicated time. (E) Lactate release and glucose consumption were evaluated in cell culture media by enzymatic colorimetric assays. Values are represented as mean ± SD, *n* = 3 (**P < 0.01 and ***P < 0.001).

To explore the regulation of glycolysis by *BRCA1*, the expression of two key glycolytic proteins, HK2 and IDH1, was measured by Western blotting (Figs. 2A–2C). In the *BRCA1* group, HK2 expression was downregulated and IDH1 expression upregulated. We analyzed respiration in MCF-7 cells by measuring pO_2_ in cell culture medium (Fig. 2D). Oxygen is consumed exclusively *via* the mitochondrial respiratory chain. Under hypoxic conditions, the MCF-7-*BRCA1* group consumed more O_2_ compared with the MCF-7-LXSN group. More glucose was consumed and more lactate released in the MCF-7-LXSN group than in the MCF-7-*BRCA1* group (Fig. 2E), indicating that *BRCA1* overexpression reduced glycolysis.

### *BRCA1* overexpression attenuated MCF-7 cell migration

**Figure 3:**
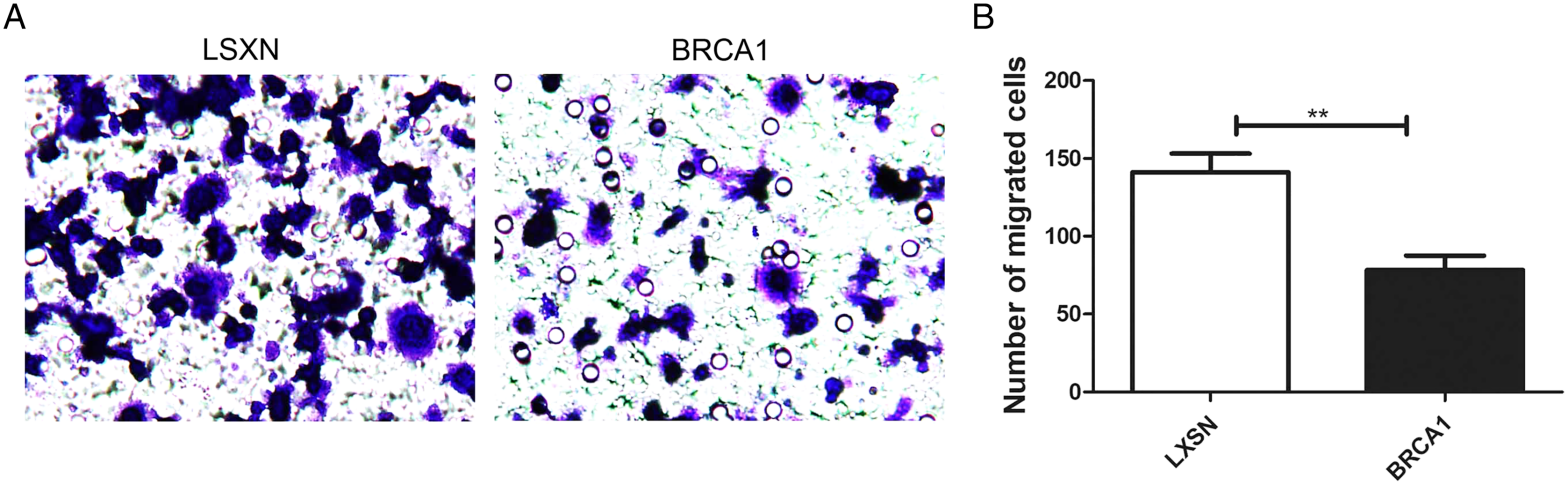
BRCA1 overexpression attenuated the migration of MCF-7 cells. (A and B) The effects of BRCA1 on migration of MCF-7 cells was measured *via* transwell method. Values are represented as mean ± SD, *n* = 3 (**P < 0.01).

The Transwell assays were used to evaluate the migration ability of MCF-7. Our results showed greater migration in the MCF-7-LXSN group compared with the MCF-7-*BRCA1* group (Figs. 3A, 3B), indicating that *BRCA1* overexpression attenuates MCF-7 cell migration.

### BRCA1 overexpression in MCF-7 cells resulted in greater sensitivity to anti-cancer agents

**Figure 4:**
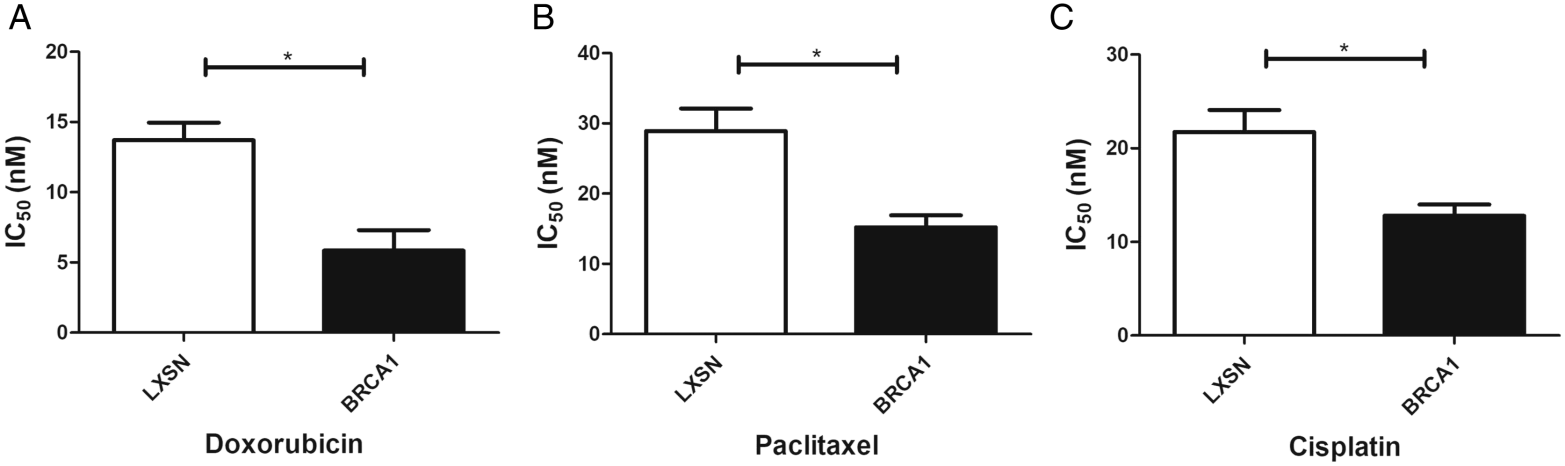
BRCA1 overexpression in MCF-7 cell shows Higher Sensitivity for anti-cancer treatment. LXSN group was demonstrated to be less response compared to BRCA1-MCF-7 group to (A) Doxorubicin, (B) Paclitaxel, (C) Cisplatin. LXSN and BRCA1 MCF-7 cells response to treatment was meassured by IC_50_ for 24h with anti-cancer agents. Values are represented as mean ± SD, *n* = 3 (*P < 0.05).

Our work aimed to clarify the role of *BRCA1* in the sensitivity to anti-cancer drugs (doxorubicin, paclitaxel, and cisplatin). *BRCA1* overexpression significantly increased the IC_50_ index for doxorubicin, paclitaxel, and cisplatin compared with MCF-7-LXSN cells, indicating that *BRCA1* promotes the sensitivity of MCF-7 cells to anti-cancer treatment (Fig. 4).

### BRCA1 overexpression reduced glycolytic flux *via* regulation of PI3K/AKT signaling

**Figure 5:**
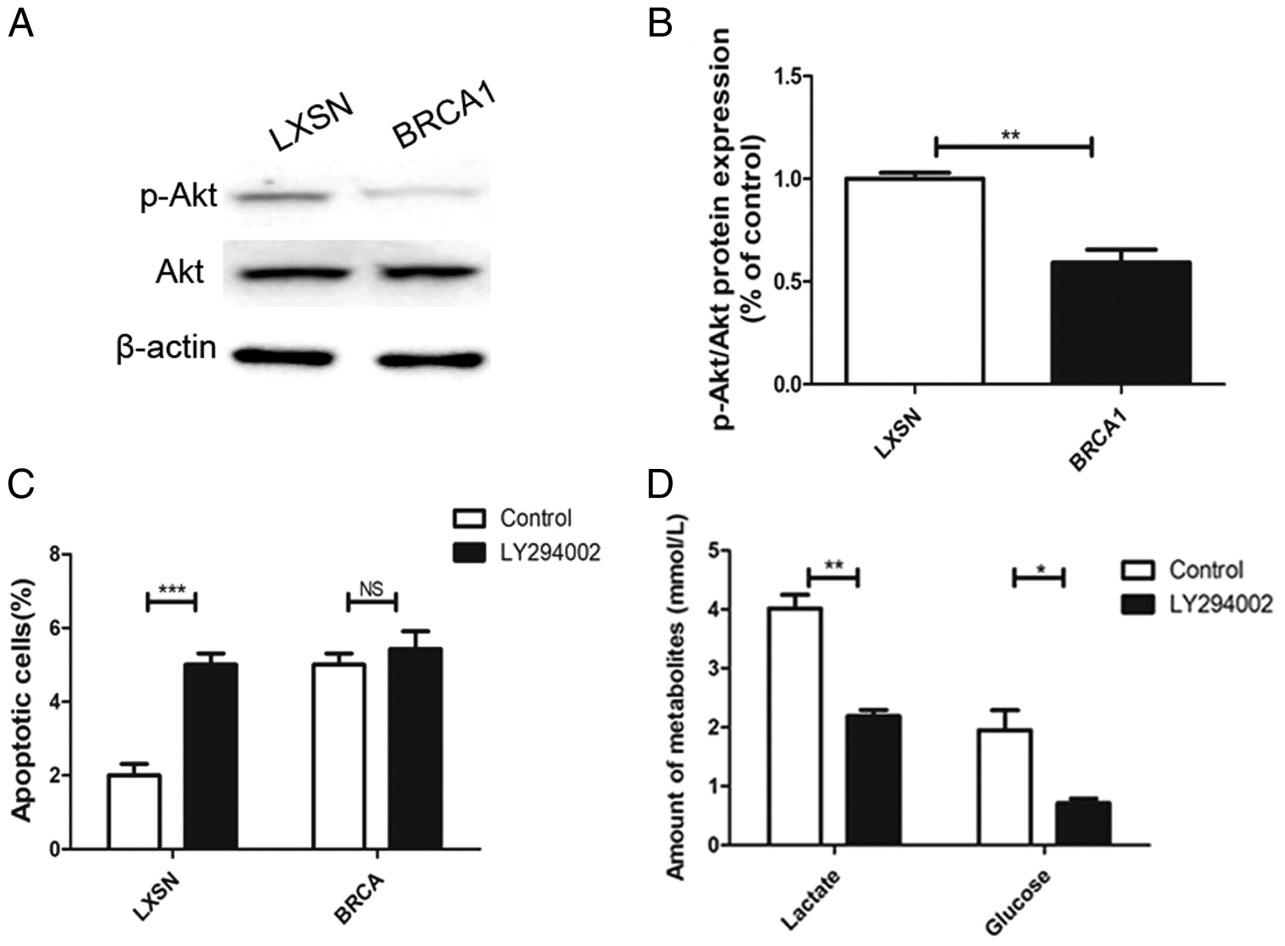
BRCA1 overexpression reduced glycolytic flux by regulation of PI3K/AKT signalling. (A and B) The PI3K/AKT signaling was measured by Western blot. (C) Number of apoptotic cells was measured at 2 days. (D) Lactate release and glucose consumption were evaluated in cell culture media. Values are represented as mean ± SD, *n* = 3 (*P < 0.05, **P < 0.01 and ***P < 0.001).

The *AKT* oncogene promotes the transcription of numerous genes encoding proteins involved in glycolytic signaling (Robey & Hay, 2009). p-AKT level was up-regulated in the MCF-7-LXSN cells than in the MCF-7-*BRCA1* cells (Figs. 5A, 5B). AKT inhibition by LY294002 increased the apoptotic MCF-7-LXSN cell population slightly, but not that of MCF-7-*BRCA1* cells (Fig. 5C). Inhibiting AKT signaling using LY294002 attenuated lactate release from MCF-7 cells. In the MCF-7-*BRCA1* group, inhibiting AKT using LY294002 had no significant effect on glucose consumption or lactate production (Fig. 5D). The results indicated that *BRCA1* overexpression contributes to regulation of the PI3K/AKT pathway, decreasing glycolytic flux.

### Depletion of PKM2 suppressed PI3K/AKT signaling

**Figure 6:**
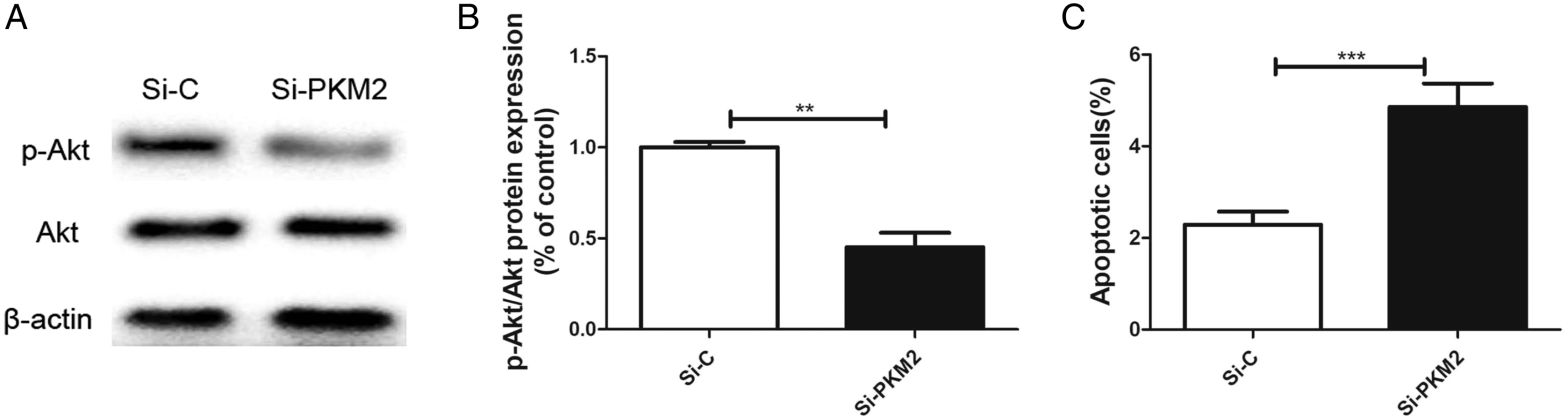
Inhibiting PKM2 activated the PI3K/AKT signalling. (A and B) The PI3K/AKT signaling was measured by Western blot. (C) Number of apoptotic cells was measured at 2 days via the Annexin V assay. Values are represented as mean ± SD, *n* = 3 (**P < 0.01 and ***P < 0.001).

Previously, we reported that *BRCA1* reduces PKM2 expression. To explore the effects of PKM2 on cancer-related signaling pathways, we used siRNA to knockdown PKM2 in MCF-7 cells. A lower p-AKT level was seen in cells transfected with PKM2 siRNA compared with control siRNA (Figs. 6A, 6B). Depletion of PKM2 resulted in increased MCF-7 cell apoptosis (Fig. 6C). All results suggested that the PKM2 is involved in apoptotic activity *via* regulation of PI3K/AKT signaling.

### MCF-7 cells were more sensitive to anti-cancer agents after depletion of PKM2

**Figure 7:**
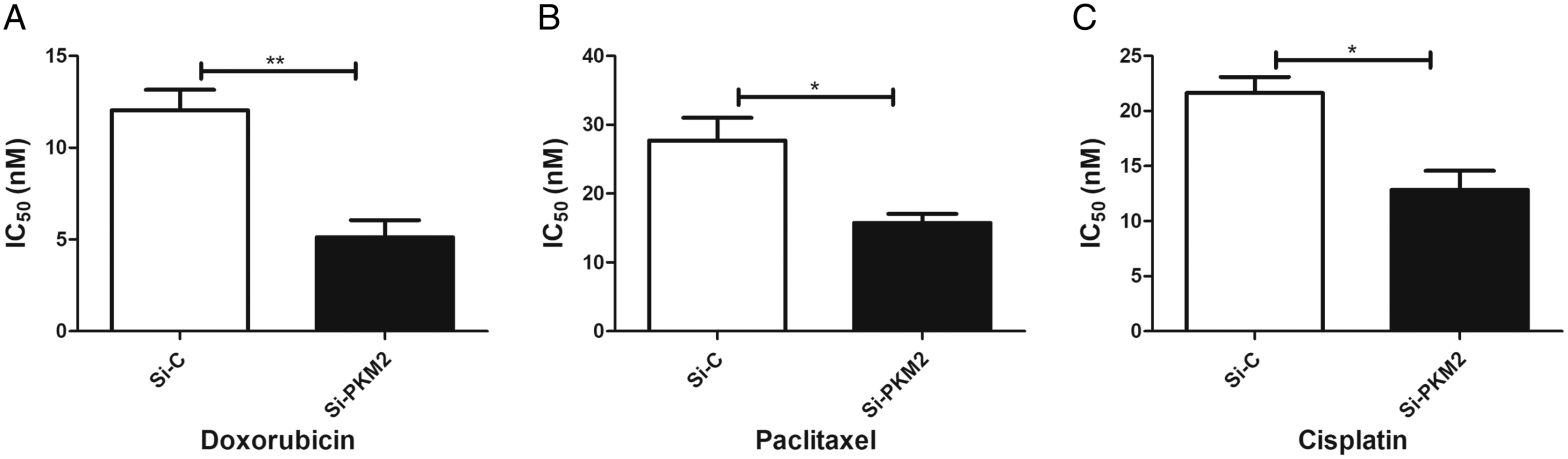
Inhibiting PKM2 in MCF-7 cell shows higher sensitivity for anti-cancer treatment. Si-C group is demonstrated to be less response as compared to Si-PKM2 MCF-7 group to (A) Doxorubicin, (B) Paclitaxel, (C) Cisplatin. Si-C and Si-PKM2 MCF-7 cells response to treatment was measured by IC_50_ for 24 h with anti-cancer agent. Values are represented as mean ± SD, *n* = 3 (*P < 0.05, **P < 0.01).

To examine the effect of PKM2 depletion on MCF-7 cells, the sensitivity to anti-cancer agents was measured in cells transfected with PKM2 siRNA. Our results suggest that siRNA-mediated depletion of PKM2 markedly reduced the IC_50_ index of doxorubicin, paclitaxel, and cisplatin, indicating that PKM2 attenuated the sensitivity of MCF-7 to anti-cancer drugs (Fig. 7).

### Depletion of PKM2 attenuated MCF-7 cell migration

**Figure 8:**
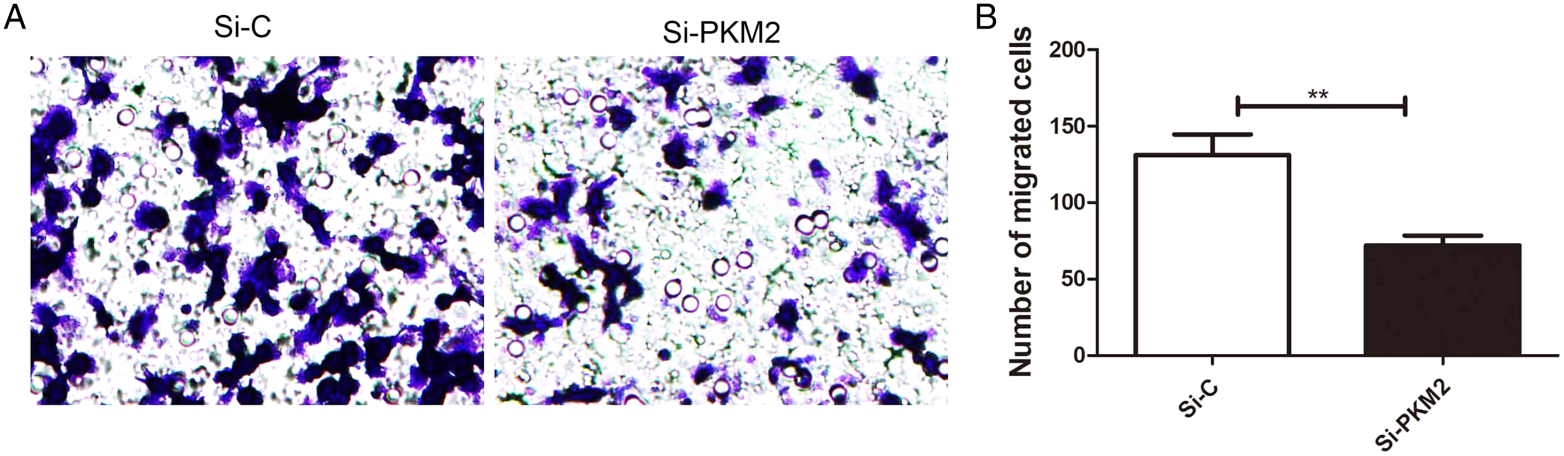
Inhibiting PKM2 attenuated the migration of MCF-7 cells. (A and B) The role of PKM2 in migration of MCF-7 cells was evalutated by transwell method. Values are represented as mean ± SD, *n* = 3 (**P < 0.01).

To examine the effect of PKM2 depletion on migration ability of MCF-7 cells, the Transwell assay was measured in cells transfected with PKM2 siRNA. Our results showed that PKM2 siRNA decreased MCF-7 cell migration compared with the control siRNA (Fig. 8), suggesting that PKM2 induces breast cancer cell migration.

## Discussion

*BRCA1* is a major breast cancer suppressor gene with a high mutation rate in hereditary breast cancer. *BRCA1* expression is reduced in sporadic breast cancer (Li et al., 2020b; Miki et al., 1994; Narod & Foulkes, 2004). This enzyme orchestrates cellular responses to stress and DNA injury. *BRCA1* is involved to DNA repair, cell cycle regulation, transcription, ubiquitination, apoptotic activity, and sensitivity to anti-cancer drugs (Yarden & Papa, 2006). In the 1920s, Warburg found that the rates of glucose uptake and glycolysis were higher in cancer cells than normal cells (Warburg, 1956). The glycolysis product pyruvate is converted mainly into lactic acid, which is discharged from cells to produce a small amount of energy (Warburg, 1956). This phenomenon is called the Warburg effect and is an inefficient way for cells to generate energy. Some researchers believe that the use of glycolysis rather than a more efficient metabolic pathway in cancer cells is closely involved in the occurrence and development of cancer (Zhang, Darshi & Sharma, 2018). Cancer cells acquire energy mainly *via* the Warburg effect or aerobic glycolysis. Reversing or weakening the Warburg effect has become a focus of cancer prevention and treatment. The PK enzyme controls the rate of the last step of glycolysis. With the lactate dehydrogenase, PKM2 enables the production of lactic acid from pyruvate, promotes aerobic glycolysis, contributes to the biosynthesis of lipids, nucleic acids and proteins, and provides favorable conditions for the proliferation and metastasis of cancer cells (Xiaoyu et al., 2018; Ye et al., 2019). PKM2 deletion in the mammary glands of a *BRCA1*-loss-driven cancer model did not postpone carcinogenesis (Israelsen et al., 2013). *HK2* is upregulated in cancer cells and is the major gene contributing to Warburg glycolysis (Privat et al., 2014). Some glycolysis suppressors targeting HK2 have been used to treat cancer (Mathupala, Ko & Pedersen, 2009). Increased glucose uptake provides energy for glycolysis in cancer cells to compensate for the lack of ATP produced by glycolysis; as a result, cancer cells produce high levels of lactic acid, thereby decreasing the pH of the cell microenvironment (Jia et al., 2018; Ye et al., 2019). The lactic acid content reflects the level of cell glycolysis occurring. We showed that *BRCA1*-MCF-7 cells expressed less PKM2 and exhibited decreased glycolysis (downregulated HK2 expression, upregulated IDH1 expression, increased O_2_ and glucose consumption, and increased lactate production) compared with the empty LXSN group. *BRCA1* transfection also contributed to enhanced apoptotic activity and decreased cell migration. Chemotherapeutic method is the most predominant strategy for treating solid tumor particularly in metastatic manner (Sharifi et al., 2014). Anti‑mitotic chemotherapy is performed as the first‑line treatment for breast cancer. Paclitaxel combined with B-tubulin and triggers apoptotic signaling *via* stabilizing microtubule and inducing cell-cycle arrest (Fabbri et al., 2006). Cisplatin interferes with DNA replication, which contributes to the death of fastest proliferating cell. One important issue regarding chemotherapeutic agents is the emergence of chemo resistance to the drug regimen which in turn reduces the efficacy of chemotherapy (Sadava & Kane, 2013). Previous study have demonstrated that reduction of *BRCA1* in breast cancer cell can increase the sensitivity of cisplatin and also lead to the resistance of anti-microtubule drugs (such as paclitaxel and vincristine) (Lafarge et al., 2001). *BRCA1* is involved in the regulation of cell cycle and apoptotic activity *via* the c-Jun N-terminal kinase signaling, suggesting that these mechanisms may be related to the resistance of cisplatin (Kennedy et al., 2004). In present work, *BRCA1* overexpression significantly increased the IC_50_ index of doxorubicin, paclitaxel, and cisplatin. These data suggest that *BRCA1* has anticancer effects by inhibiting PKM2-mediated glycolysis.

Several pathways are likely involved in the effects of *BRCA1* on glycolysis. One is AKT signaling. *BRCA1* inactivates the oncoprotein AKT (Xiang et al., 2008), which induces glycolysis *via* multiple mechanisms, including promoting the expression and activation of hexokinases (*e.g*., HK2) (Robey & Hay, 2009). The present data suggested that *BRCA1* overexpression reduces AKT phosphorylation. The use of LY294002 to inhibit AKT signaling resulted in decreased lactate release from MCF-7 cells. Transfection of *BRCA1* in MCF-7 cells led to reduced glycolysis *via* regulation of the PI3K/AKT pathway.

Genetic research on cancer susceptible constitutions and high-throughput sequencing of cancer cell genomes have shown that metabolic enzyme mutations are critical for the development of cancer (Qi et al., 2018). PKM2 is a key enzyme in glycolysis, which supplies energy for the synthesis of macromolecules required for cell proliferation, such as proteins, lipids, and nucleic acids. PKM2 catalyzes the conversion of phosphoenolpyruvate to pyruvate. Four PK isozymes are known: PKL, PKR, PKM1, and PKM2. Of these, PKM2 is up-regulated in most cancer cells and acts independently of the cancer tissue source (Mazurek, 2011). PKM2 affects the occurrence and development of cancer. Christofk et al. (2008) confirmed that a high PKM2 level promotes aerobic glycolysis in cancer cells. Inhibiting PKM2 using shRNA in a human lung cancer cell line reduced its carcinogenicity, confirming the important role of PKM2 in cancer cell growth (Christofk et al., 2008). Microarray analysis also showed that PKM2, the rate-limiting enzyme of glycolysis, was one of the most upregulated genes in cancer cells (Altenberg & Greulich, 2004). Recent studies have found that the PKM2 level is related to multidrug resistance (Israelsen et al., 2013). For example, cancer cells exposed to oxaliplatin showed upregulated PKM2 expression, which led to oxaliplatin-resistant cancers. Therefore, PKM2 can also be used as an indicator of resistance to anti-cancer drugs such as oxaliplatin (Martinez-Balibrea et al., 2009). PKM2 is a very sensitive cancer marker and may become an important target of anti-cancer drugs. The present work showed that depleting PKM2 using siRNA markedly reduced the IC_50_ index of doxorubicin, paclitaxel, and cisplatin. Depleting PKM2 also suppressed PI3K/AKT signaling and increased MCF-7 cell apoptosis. Transwell migration assays showed that PKM2 siRNA attenuated the migration of MCF-7 cells compared with control siRNA. Therefore, PKM2 may become a new marker for diagnosing cancer, evaluating treatment efficacy, and determining prognosis.

The limitation is that the details of the interaction between *BRCA1* and PKM2 needs further study, and we will continue to explore the mechanism of PKM2 in depth.

## Conclusion

Our results suggest that *BRCA1* overexpression inhibits the Warburg effect, decreases cancer cell growth and migration, and enhances sensitivity to anti-cancer treatments *via* downregulation of PKM2 regulated by PI3K/AKT signaling. These novel metabolic effects of *BRCA1* are a potential mechanism by which it inhibits breast cancer.

## Supplemental Information

10.7717/peerj.14052/supp-1 Supplemental Information 1 Raw data.

10.7717/peerj.14052/supp-2 Supplemental Information 2 Raw numeric data.

## Additional Information and Declarations

### Data Availability

The following information was supplied regarding data availability: The raw data is available in the Supplemental Files.
