# Supplementary material for: BRCA1 overexpression attenuates breast cancer cell growth and migration by regulating the pyruvate kinase M2-mediated Warburg effect via the PI3K/AKT signaling pathway
Source: PeerJ. 2022 Sep 28;10:e14052. doi: 10.7717/peerj.14052 (PMC9526413; doi:10.7717/peerj.14052)

Figure1

BRCA-1
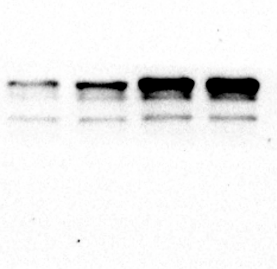
PKM2
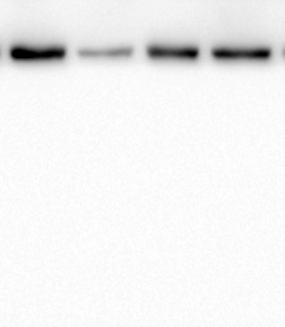
B-actin
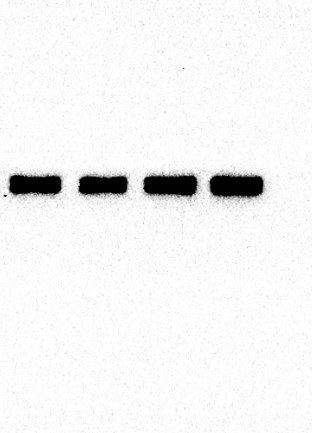


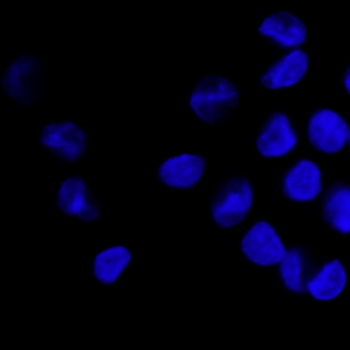

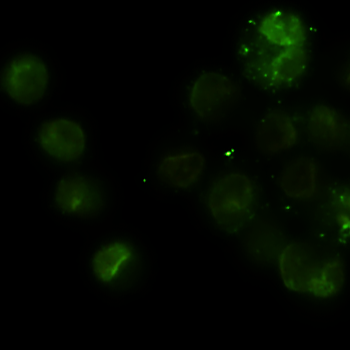

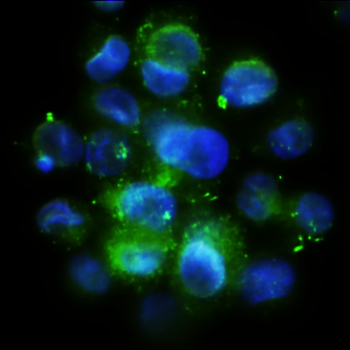

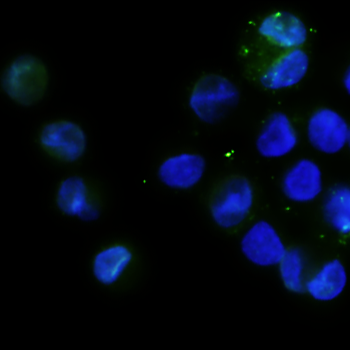

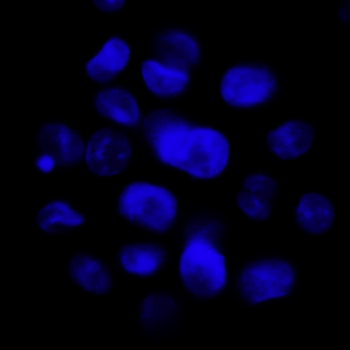

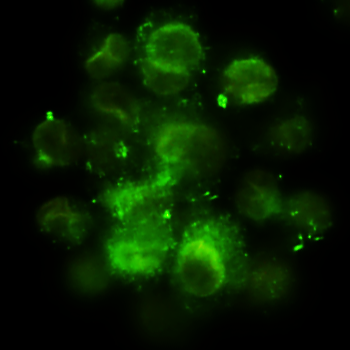


Figure2

HK2
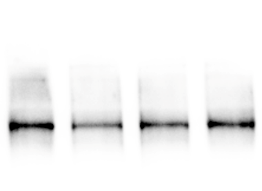
IDH1
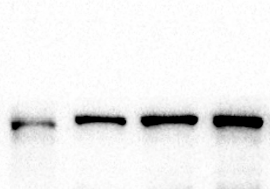
b-actin
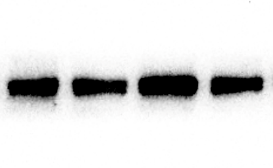


Figure3


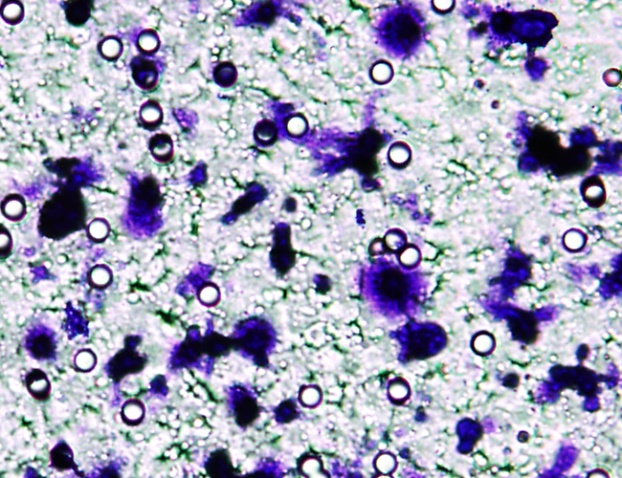

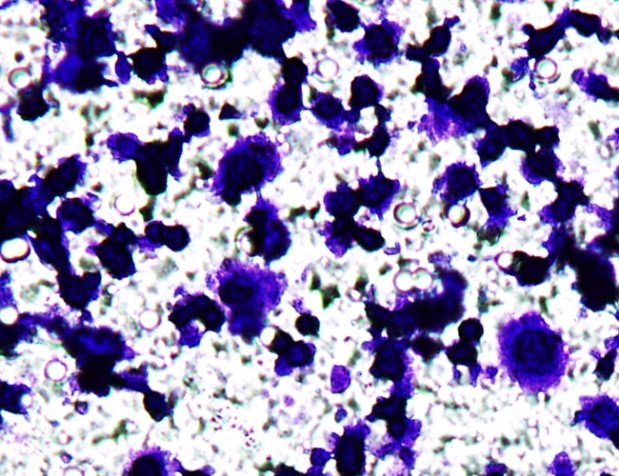


Figure5

p-akt
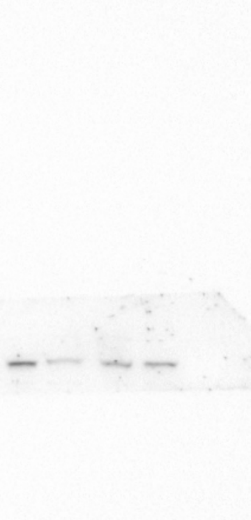
t-akt
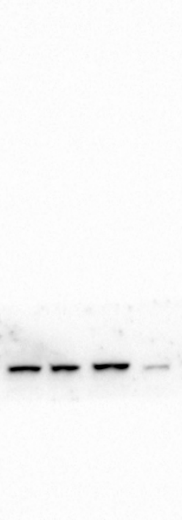
b-actin
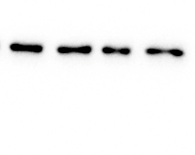


Figure6

pakt
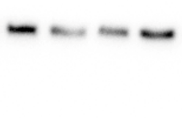
t-akt
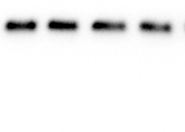
b-actin
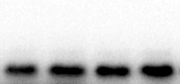


Figure8


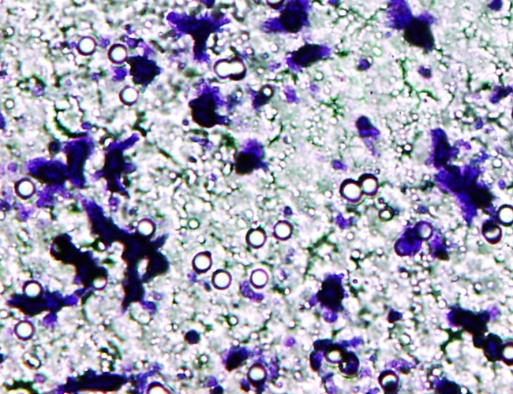

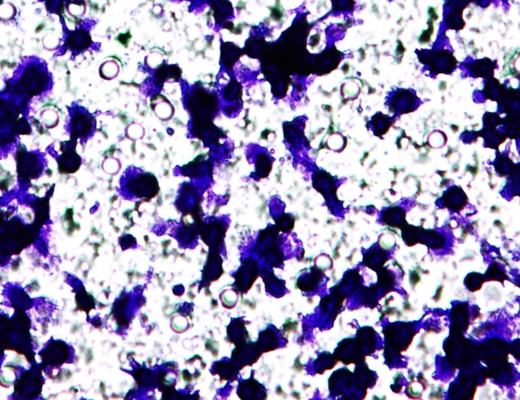

Supplement: Supplemental Information 1 [file peerj-10-14052-s001.docx]
